# Fever

**DOI:** 10.1100/tsw.2010.50

**Published:** 2010-03-16

**Authors:** Tamas Bartfai, Bruno Conti

**Affiliations:** Molecular and Integrative Neurosciences Department, The Scripps Research Institute, La Jolla, CA, USA

**Keywords:** fever, antipyretic, interleukin, temperature, hypothalamus

## Abstract

Measurement of body temperature remains one of the most common ways to assess health. An increase in temperature above what is considered to be a normal value is inevitably regarded as a sure sign of disease and referred to with one simple word: fever. In this review, we summarize how research on fever allowed the identification of the exogenous and endogenous molecules and pathways mediating the fever response. We also show how temperature elevation is common to different pathologies and how the molecular components of the fever-generation pathway represent drug targets for antipyretics, such as acetylsalicylic acid, the first “blockbuster drug”. We also show how fever research provided new insights into temperature and energy homeostasis, and into treatment of infection and inflammation.

## INTRODUCTION

Can a frog or a snake run a fever? Is the ramp fever (stage fright) a real fever like that in malaria? Did Moses see the burning bush in febrile hallucination since malaria was endemic along the Nile? Is fever helping to fight infection? Should fever be treated or permitted? These are but a few of the questions we have received in the past 27 years from friendly, but incredulous, colleagues who have not understood why a well-trained scientist would "fiddle with fever".

Despite the fact that fever was used throughout history, and still continues to be used, as the most common noninvasive measure of disease, its study is considered by many to be useless. Among the arguments by skeptics towards the relevance of studying fever was the fact that, as pointed out by Carl Reinhold August Wunderlich, "Fever is not a disease, but rather the response of the body to a disease." In addition, since fever can now be controlled effectively with a variety of inexpensive and safe antipyretics, the "battle against fever" from a therapeutic, pharmacological perspective can be considered won. As is the case when remarkable achievements are ultimately taken for granted, it is often forgotten that fever was a serious health concern until effective antipyretics, such as acetylsalicylic acid, paracetamol (acetaminophen), and ibuprofen, were found. The introduction of antipyretics had a large impact well beyond medicine and science. Acetylsalicylic acid (aspirin), the first synthetic antipyretic, was originally tested in a "one-patient clinical trial" and gained worldwide popularity during the Spanish flu pandemic before becoming an object of the peace terms of the First World War, forcing Bayer to give up its trademark.

Despite the general attention by the medical community to the benefits of reducing fever, Julius Wagner-Jauregg received the 1927 Nobel Prize in Physiology and Medicine for his malaria-based fever therapy "of the general paralysis of the insane" caused by syphilis. Fever therapy has been replaced by antibiotics in the treatment of syphilis, and the mechanisms of fever are still being elucidated and the question of whether fever is beneficial or detrimental to the organism remains unanswered.

Fever can be described as an elevation of the central thermoregulatory set point, largely achieved by disinhibiting thermogenesis. This definition resulted from over 100 years of research during which the molecules mediating the fever response have been identified. Some were discovered only recently by groups investigating not strictly fever, but temperature homeostasis in general.

Fever, together with torpor and hibernation (these two limited to a few species) represent a controlled deviation of organisms from the otherwise finely maintained temperature homeostasis. In homeotherms, including humans, body temperature is tightly controlled with circadian fluctuations of only 1.5˚C about its normal value of 36.5˚C or 279.5˚K. Even fever is characterized by a temperature increase of similarly small magnitude (2–4˚C). Since homeostatic mechanisms can be more easily investigated when the systems they maintain are brought far from their equilibrium, fever represents one experimental model to investigate the biology of temperature regulation. The implications of such an investigation stretch from understanding homeothermia, a property that allowed the colonization of biotopes with very different ambient temperatures, to understanding the contribution of temperature to energy homeostasis and its influence on lifespan. Finally, important medical applications may be found in both raising and lowering core body temperature (CBT).

In this review, we summarize the efforts of the first pioneers of fever research, describing how strong were the foundations they set for the future of fever research despite the technical and experimental limitations of their times. We also describe how fever research was affected by the chemical synthesis of the stable form of acetylsalicylic acid, and how the introduction of new technologies and experimental models allowed for the identification of molecules mediating the fever response, including exogenous as well as endogenous pyrogens.

## A (VERY) BRIEF HISTORY OF FEVER RESEARCH

The classical literature on fever studies from France, Germany, the Austro-Hungarian Empire, the U.K., and later from the U.S. is a marvel of conceptual foresight and careful experimentation in physiology. It was carried out by using thermometers as well as early calorimeters, i.e., large, double-walled metal boxes with water between the walls, in which heat dissipation in experimental models were studied with great precision, sometimes for weeks[1,2]. The animal of choice for these studies was the rabbit because of the large reproducible fever response it exhibited (2–4˚C), because of the large body mass important in calorimetry, and finally for its patient ability to sit in a box for weeks. The scientific competition in this field was fierce and within 2–3 years, any published data deemed important was repeated in at least two laboratories and was confirmed or, often in polite but strong terms, rejected.

There are beautiful monographs over fever research during this period that detail the controversies that mostly centered on the variability of the preparations (i.e., composition and purity) used to elicit fever, as well as the differences in strain, size, or age of the rabbits studied. One particularly insightful and enjoyable example is the *General Pathology of Fever*, the Cartwright lectures by Welch (1888)[3]. The variability of results was accentuated by the very small number of animals in each study and the fact that different laboratories did not utilize the same strains of rabbits.

Despite the technical limitations of the late 1800s, scientists like Ott, Aronsohn, Sachs, Liebermeister, and Lavoisier described fever response quite exhaustively and formulated most of the important questions on the subject, also providing some answers. For instance, they concluded that fever was evoked by a wide variety of substances and that it was a highly coordinated, stereotypic response regulated by the brain. They also determined that certain experimental brain lesions of the anterior hypothalamus led to "fever" in the absence of injecting any pyrogen, an observation that led to the idea that fever was a *disinhibition* in which the hypothalamus acted to release an inhibition on peripheral heat production[4,5]. Indeed, the last 10 years of neuroanatomical and physiological work in establishing the neuronal links between the hypothalamus and the heat-producing brown adipose tissue[6,7] have proven that the hypothalamic control is inhibitory and that fever ensues when the inhibition is released by pyrogen action on neurons of the anterior hypothalamic thermoregulatory center[8], fully proving Ott's hypothesis.

Some of the limitations that challenged the early days of fever research were overcome when mice were introduced as an animal model[9]. This may appear as a very simple achievement if it was not for the fact that mice do not always develop an easy-to-measure fever response. Unlike rabbits that have a larger mass/surface ratio than mice, heat production is already running at such a high rate in small animals that at room temperature, the fever response in terms of elevated temperature is hard to observe and measure. It was not until the early 1980s, with the introduction of the use of thermoneutral rooms for rodents (rats 25˚C, mice 28˚C), that fever response as a regulated rise in CBT could be easily followed (Fig. 1). Mice, as an experimental model, offered several advantages over the use of rabbits. They were more affordable and the number of animals that could be investigated per group went from an average of 2–3 per study to 6–15 or more, thus increasing the statistical significance of the measurements. Mice eventually became available in pure inbred strains and the same strain could be used by different groups all over the world, thus reducing the genetic differences among experiments performed in different laboratories.

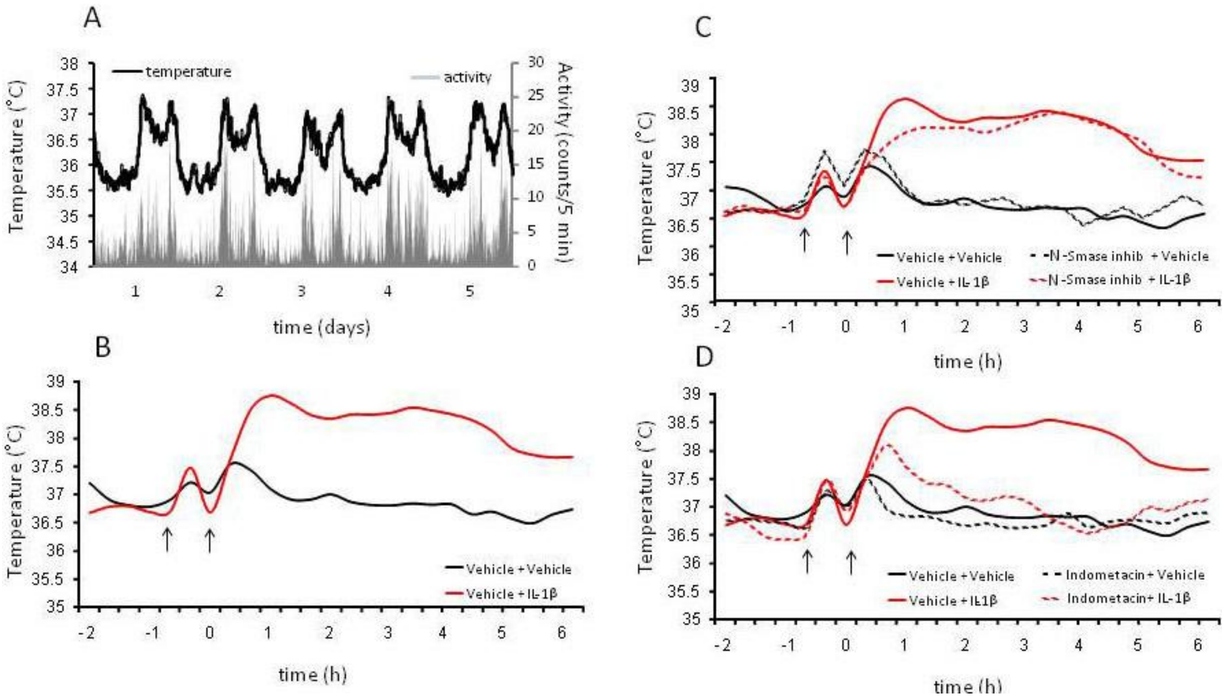

**FIGURE 1.** Representative profiles of circadian temperature and fever response in mice. (A) Profile CBT (top) and motor activity (bottom) over 5 days of continuous recording; (B) 24-h temperature profile of mice injected with vehicle (saline) and subsequently with the endogenous pyrogen interleukin-1β (IL-1β), showing a stress-dependent modest temperature elevation following vehicle injection and the fever response after lipopolysaccharide (LPS) treatment; (C) N-Smase attenuates the first, fast-acting phase of the fever response to IL-1β; (D) indometacin inhibited the second, long-lasting fever response to IL-1β.

Eventually, two additional, important technological advancements allowed the research on fever to move forward considerably: the use of radiotelemetry[10,11] and the development of transgenic mice. An adaptation of military technology originally developed to track submarines, radiotelemetry was introduced into fever research in the mid-1980s, allowing the recording of CBT continuously without disturbing the animals. This technology also allowed researchers to overcome an important limitation of the use of rectal thermometers: the stress-dependent fast and steep increase of CBT observed while handling the animals, which often provided false CBT values. Continuous recording also allowed for the precise recording of the normal circadian fluctuation of CBT; in addition, radiotelemetry also evolved, allowing for the measurement of motor activity, an important contributor to CBT.

Transgenic mice, in which the function of one or another protein is prevented or increased by genetic techniques, permitted the final identification of the molecules participating in the fever response. For instance, the use of transgenic mice was essential in delineating pathways, such as the Toll signaling pathway, coupling exogenous and endogenous pyrogens.

Despite the availability of antipyretics, clinicians remained firm in their insistence on taking the temperature of the hospital patient at least three times a day, and used the rise and decline of fever as an important indicator of pathophysiology. In the absence of measurements of bacterial or viral proliferation, this was, and remains even today, a great diagnostic tool to follow the course of infection and inflammation, and the success or failure of the immune system to fight it. However, fever alone rarely revealed the nature of a pathology conclusively. Thus, another important question in fever research was: How was it possible that a variety of chemically and biologically diverse substances (from inorganic school chalk to biological substances such as bacterial and viral components of protein, lipid, lipoprotein, and of nucleic acid nature) could cause the very same fever response? This question would not find an answer until "the endogenous pyrogen" was identified[12,13].

## PYROGENS

Pyrogens are, by definition, substances that can induce fever. They can be distinguished as endogenous or exogenous pyrogens[13]. This distinction is fundamental in understanding fever as a common response to different stimuli – bacterial, viral, or emotional – and emphasizes the importance of the recent discovery of the Toll signaling pathway that provides a molecular link between the multitude of exogenous and endogenous pyrogens, which they induce[14,15].

### Exogenous Pyrogens and TLRs

The most popular exogenous pyrogen utilized in the study of fever was the bacterial lysate from Gram-negative bacteria[16]. The key pyrogenic component of the lysate was determined in fractionation experiments to be the bacterial cell wall component lipopolysaccharide (LPS) (Fig. 2). The existence of a hypothetical LPS receptor was postulated and sought as a putative drug target for an antibacterial agent. Decades of studies carried out using radiolabeled LPS yielded several diverse, but inconclusive, ill-defined, candidate receptors as well as binding proteins. Finally, a protein that recognized and bound LPS with high affinity was identified in the Beutler laboratory by positional cloning in the "LPS-resistant mouse" of a gene with a point mutation that conferred resistance to LPS. The gene encoded a single transmembrane domain receptor belonging to the Toll-like receptor (TLR) family and was eventually named TLR4. This receptor is activated upon binding the complex LPS-LPS binding protein[17]. This discovery was rapidly followed by the identification of other TLRs that recognize other specific bacterial or viral components, including DNA and RNA[15,17,18]. TLRs can activate the signal transduction cascade in macrophages and other immune competent cells leading to the nuclear factor kappa B (NF-κB)–dependent synthesis of cytokines, including interleukin-1 (IL-1), the tumor necrosis factor (TNF), as well as the rate-limiting enzyme for the synthesis of prostaglandins, cyclo-oxygenase 2 (COX2) (Fig. 3).

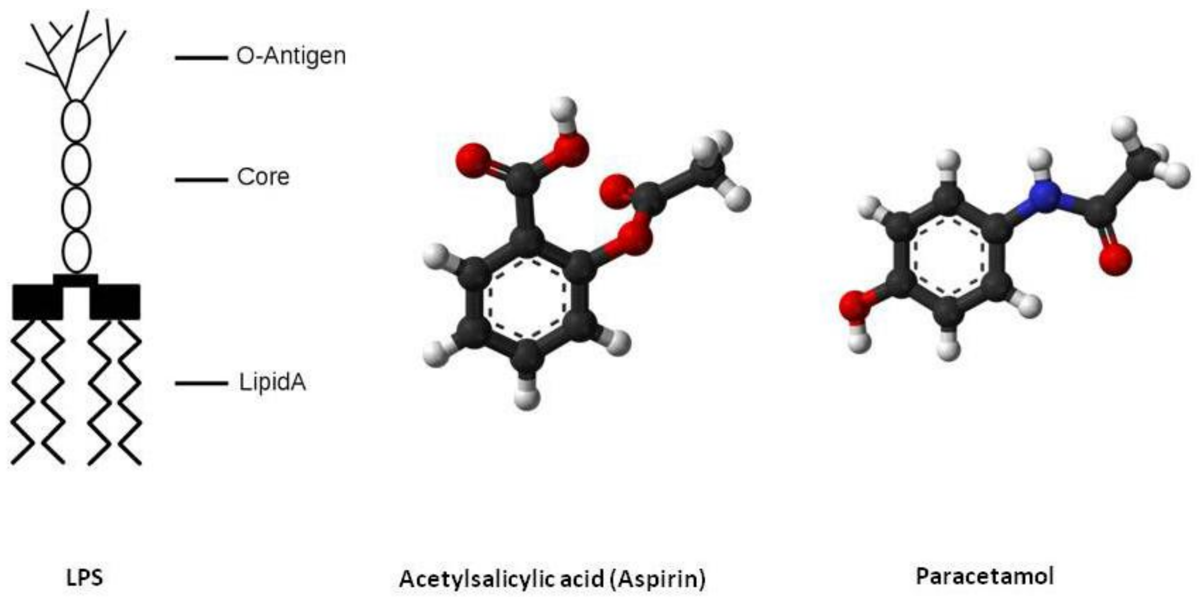

**FIGURE 2.** Exogenous pyrogens and antipyretic drugs. Molecular models of the exogenous pyrogens LPS and the antipyretic medications acetylsalicylic acid (aspirin) and paracetamol.

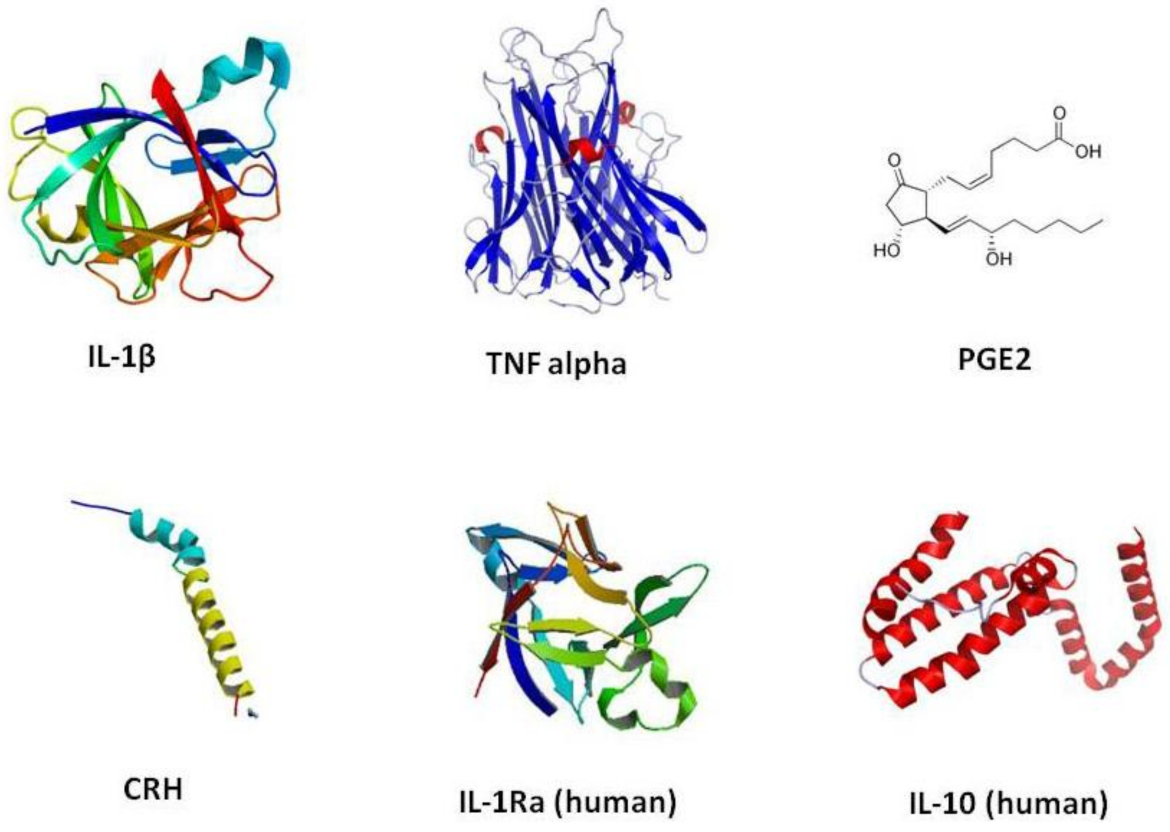

**FIGURE 3.** Endogenous pyrogens and antipyretics. Molecular structure of endogenous pyrogens IL-1β, TNF, prostaglandin E2 (PGE2), corticotropin releasing hormone (CRH), and two endogenous antipyretics involved in the termination of the fever response, the IL-1 receptor antagonist (IL-1RA) and IL-10. All structures were obtained from PDB (http://www.rcsb.org) and rendered using Pymol (pymol.sourceforge.net).

## Endogenous Pyrogens and the IL-1 System

The parallel studies of the "endogenous pyrogen"[13] and of "the lymphocyte activating factor"[19] resulted in the surprising finding that these two important factors were the same protein molecule: IL-1. IL-1 is an important intercellular messenger between cells of the immune system, including macrophages and B and T lymphocytes, but it is also a stimulator of COX2 biosynthesis.

Barely detectable during the uninfected state or in the absence of inflammation, the circulating level of IL-1 increases 20- to 100-fold 30–90 min after TLR activation by virus or bacteria. Thus, via IL-1, the fever response is a hallmark of infection. Although other endogenous pyrogens exist in addition to IL-1, e.g., TNF and IL-6, we will treat in detail only the IL-1 system that remains the most relevant for understanding fever, while the other endogenous pyrogens play important roles in the inflammatory sequelae in diseases like psoriasis and rheumatic arthritis.

The IL-1 system is composed of two agonist type ligands, the membrane-bound IL-1α and the soluble IL-1β, secreted upon maturation (proteolytic cleavage) of a precursor by caspase 1, a cystein endoprotease. In addition to IL-1α and IL-1β, there exist three isoforms, two intracellular and one secreted, of the IL-1 receptor antagonist (IL-1RA). These ligands bind to a heterodimeric, cell surface receptor composed of an IL-1R1 and the IL-1 receptor accessory protein (IL-1RAcP). A decoy soluble receptor (IL-1R type 2) also exists. Interestingly, the IL-1R1 and the IL-1RAcP are both members of the Toll receptor family[14], as they express a protein-protein interaction domain in their intracellular portion called the TIR (Toll receptor interacting) domain[20]. Thus, in its signaling, the endogenous pyrogen IL-1 utilizes the same molecular domains (TIR domains) and the same signaling pathway (activation of NF-κB), involving the same proteins, as the exogenous pyrogens. This indicates that during early infection, the exogenous pyrogens that activate Toll receptors and the newly formed endogenous pyrogens synergistically contribute to the exogenous pyrogen–initiated fever. It is only when the exogenous pyrogens are eliminated by the immune system, and the endogenous antipyretics are synthesized and released and can compete with the endogenous pyrogens, that fever can subside.

Several hereditary diseases that are associated with infection-independent fever have been shown to be caused by imperfect control of IL-1β production and release[21], including Familial Mediterranean Fever and Mucke-Wells Disease[22]. Others are instead associated with derailed TNF signaling, like the TNF receptor–associated period syndrome[23], although also in this case the administration of IL-1RA and IL-1β neutralizing antibodies block the fevers. Another remarkable example is febrile seizure unresponsive to traditional anticonvulsants and experienced by up to 7% of infants[24]. Recent research suggested that IL-1β contributes to this disorder by reducing seizure thresholds, thus acting as a proconvulsant[24,25]. A similar mechanism is also believed to mediate fever and seizure following head trauma, as activated microglia in the damaged brain become a rich source of IL-1β. IL-1RA is today tested as a potential drug in these cases of febrile seizures as it is a potent anticonvulsant[25] in seizures induced chemically, or in the case of head trauma and stroke[26]. The efficacy of IL-1RA in reducing febrile seizures, and trauma-related seizure and fever, also provides an example of the role of IL-1RA as an endogenous antipyretic and anti-inflammatory.

IL-1RA synthesis is, in fact, potently stimulated by IL-1β and other endogenous pyrogens, and raising the level of IL-1RA serves to terminate the pyretic signaling of IL-1β itself. Other molecules with similar antipyretic activity include the IL-1 type 2 nonsignaling decoy receptor, as well as members of other cytokine families participating in the modulation or ending of the inflammatory response, such as the decoy receptor for TNF and the anti-inflammatory, antipyretic-like IL-10, respectively. The existence of an endogenous high-affinity agonist and antagonist that competes for binding and activation of the same signaling receptor strongly underlines how narrowly the fever response is regulated.

## Prostaglandins

Exogenous pyrogens can induce the production of the endogenous pyrogens via activation of Toll signaling, and both endogenous and exogenous pyrogens can stimulate the synthesis of prostaglandins (PG). The role of PG, particularly of PGE2, in mediating fever is now well documented[27]. A large body of literature investigated the pharmacological action of PGE2 following both peripheral and central administration[28], as well as their endogenous release during fever[13]. In addition, it was shown that the antipyretic action of aspirin is due to its ability to inhibit cyclo-oxygenase (1 and 2), the rate-limiting enzyme in PGH and PGE2 synthesis, thus displaying antipyretic properties that are independent of the type of exogenous pyrogen causing the fever response[29,30]. These findings contributed to the designation of PGE2 as the ultimate endogenous pyrogen[31].

There exist at least four subtypes of PGE2 receptors, EP1–4, expressed in the hypothalamus. All belong to the seven-transmembrane domain, G protein-coupled receptors[31,32,33]. Unfortunately, despite their great potential therapeutic utility, no prostanoid receptor subtype–selective antagonists exist for the four prostanoid receptors, although there are some receptor subtype–selective agonists[34]. Thus, assigning which prostanoid receptor subtype(s) is involved in PGE2-mediated fever was accomplished by use of transgenic mice, displaying null mutations of the individual EP1-2-3-4 receptors. These studies showed that in fever, the key role is played by the EP3 receptor, and to a lesser degree, the EP1 receptor subtype[35]. Finally, recent studies have shown that the PGE2 occupancy of the EP3 and EP1 receptor is responsible for the slower-developing, long-lasting portion of the fever response, between 30 min and 3 h after the application of an exogenous pyrogen or after the application of IL-1, with the IL-1–induced synthesis of ceramide being responsible for the rapid fever response that precedes the PGE2-mediated portion[36].

How PGE2 via EP1 and EP3 eventually changes the “hypothalamic set point”, thus triggering the fever response, is yet to be determined and needs to be investigated at the cellular and molecular level. The most relevant information in this respect comes from electrophysiological studies showing that PGE2 increased the thermosensitivity of warm sensitive neurons via EP3R (see below).

## NEURONAL NETWORK, CELLULAR AND MOLECULAR CONTROL OF FEVER

In 1870, Ott clearly stated that the coordination of heat conservation as observed by cold extremities at the beginning of the fever response, and the heat production by nonshivering and shivering thermogenesis that follows, are accomplished by coordinated neuronal signals from the anterior hypothalamic region of the brain, a conclusion reached by systematic surgical lesions of different brain regions while measuring fever response.

The maintenance of a constant CBT as well as regulated increase during fever assumes the existence of a thermoregulatory network activity. In the anterior hypothalamus, the preoptic area (POA) is considered the most important region for temperature homeostasis. Since this region can sense local temperature changes (blood flow temperature) and activate the necessary thermoregulatory responses required for the maintenance of a constant CBT, it was hypothesized to contain the “central thermostat”. The chemical neuroanatomy of the thermostat is multilayered. It uses *heat* as a diffuse, paracrine signal affecting many cells in a close environment; it uses rapidly acting *small signal substances*, such as glutamate and GABA, serotonin, acetylcholine, and adrenaline (molecular weight <200 Da), which are synthesized by cytosolic enzymes; but it also uses a multitude of *peptide transmitters* (MW 500–5000 Da) released from the same neurons and *lipid mediators* like PGE2 and ceramide .

In the past decade, work carried out by Caterina and Julius[37] elegantly demonstrated that peripheral temperature sensitivity is mediated by transient receptor potential (TRP) channels[38]. These ion channels respond to a broad spectrum of temperature changes (0–50°C) and their identification showed beautifully how temperature changes, via activation of temperature-sensitive ion channels, can result in neuronal activity. However, these TRP channels do not appear to be responsible for the central mechanisms of thermoregulation. Although the vanilloid receptors or capsaicin action was shown in POA neurons, a decade earlier than in the periphery, their presence does not explain the behavior of the central thermostat[39].

Work aimed at understanding the nature and the mechanisms of action of the central thermostat are focused on temperature-sensitive hypothalamic neurons found in the POA of the hypothalamus[40,41]. These specialized cells can be distinguished as warm or cold sensitive, as their firing rate changes dramatically with small temperature elevation or reduction (1–3°C). They can sense local temperature changes in the hypothalamus and its blood flow[42], but also integrate information about skin temperature[6] and about the nutritional state of the body, sensing, for instance, insulin and adiponectin levels. It was also shown that they may integrate the effects of emotional states on temperature, i.e., ramp fever and other stress-induced hyperthermic responses[43].

Warm-sensitive neurons (WSN) are more easily studied than cold-sensitive neurons (CSN), primarily because of their relatively higher number in the POA (10–15% vs. 0.5–1%). POA neurons are defined as WSN if the temperature-dependent increase in their firing rate is greater than 0.8 Hz/°C[44]. In WSN, a 2–3°C increase of local temperature can produce a two- to fivefold increase in firing rate (changes far larger than predicted by the Arrhenius law), suggesting that a set of coupled reactions must participate in producing the changes in membrane potential and firing rate. These changes are too remarkably large to be simply explained as due to increased rates of leak currents occurring in every cell when temperature is increased.

Our knowledge of how heat is sensed by the WSN is extremely rudimentary and comes from studies carried out *in vivo*[45] and later in tissue slices[46]. Both systems have been utilized in the attempt to determine whether warm sensitivity is a property of the thermostat *network* or whether it is a *cellular* property. WSN could be found invariably in slices as well as in embryonal-dissociated cultures after E13[47]. In addition, after we blocked all synaptic signaling in the culture of POA neurons, warm sensitivity was a property that remained, indicating that it was a cellular and not a “network-dependent” property.

Thus, it was reasonable to assume that a comprehensive molecular characterization of the WSN could reveal the molecules mediating thermosensitivity in these cells.

Such characterization is ongoing in our laboratory, where adult WSN are identified electrophysiologically from slices of anterior hypothalamus and their content aspirated. The RNA of single WSN is reverse transcribed into cDNA, the first step in a series of reactions ultimately leading to the linear amplification of the RNA. This technique, developed by James Eberwine[48], allows the collection of sufficient amounts of nucleic acids from a single WSN, permitting the comprehensive investigation of the transcriptome. We are now in the process of validating some of the transcripts of encoding receptors and ion channels that may be involved in the fever response. We tested and successfully validated some specific receptors found to be expressed in WSN by measuring the effects of their ligands on CBT. For instance, we found transcripts for adrenomedulin and bombesin receptors, and showed that local injection of their neuropeptide ligands in the mouse POA induced hyperthermia and hypothermia, respectively. Thus, adrenomedullin was identified as a novel endogenous pyrogen and bombesin as an endogenous hypothermic (or antipyretic) agent, confirming an early observation by Hori et al.[43].

We hope that in the near future, our ongoing effort will lead to identification of the molecules that confer WSN with the ability to respond to temperature changes with changes in neuronal activity. This would open new ways to enable not only the modulation of the fever response, but also the pharmacological regulation of metabolic rate and rate of aging that is linked to it.

To contribute to the coordination of CBT regulation as well as to fever response, WSN are elements of a neuronal network schematically represented in Fig. 4. The scheme shows the pivotal role that the WSN play in integrating the temperature signals from the skin and deep body, and the metabolic and stress signals, including the pyrogens. In addition, it emphasizes the importance of the inhibition of thermogenesis by hypothalamic neurons as an important central brake, as postulated by Ott in 1870 when describing that fever represents a disinhibition mechanism. For instance, cold signals from the periphery produce an inhibition of the WSN and, subsequently, a disinhibition of heat production. Pyrogens acting on the same neurons can evoke a similar disinhibition of thermogenesis.

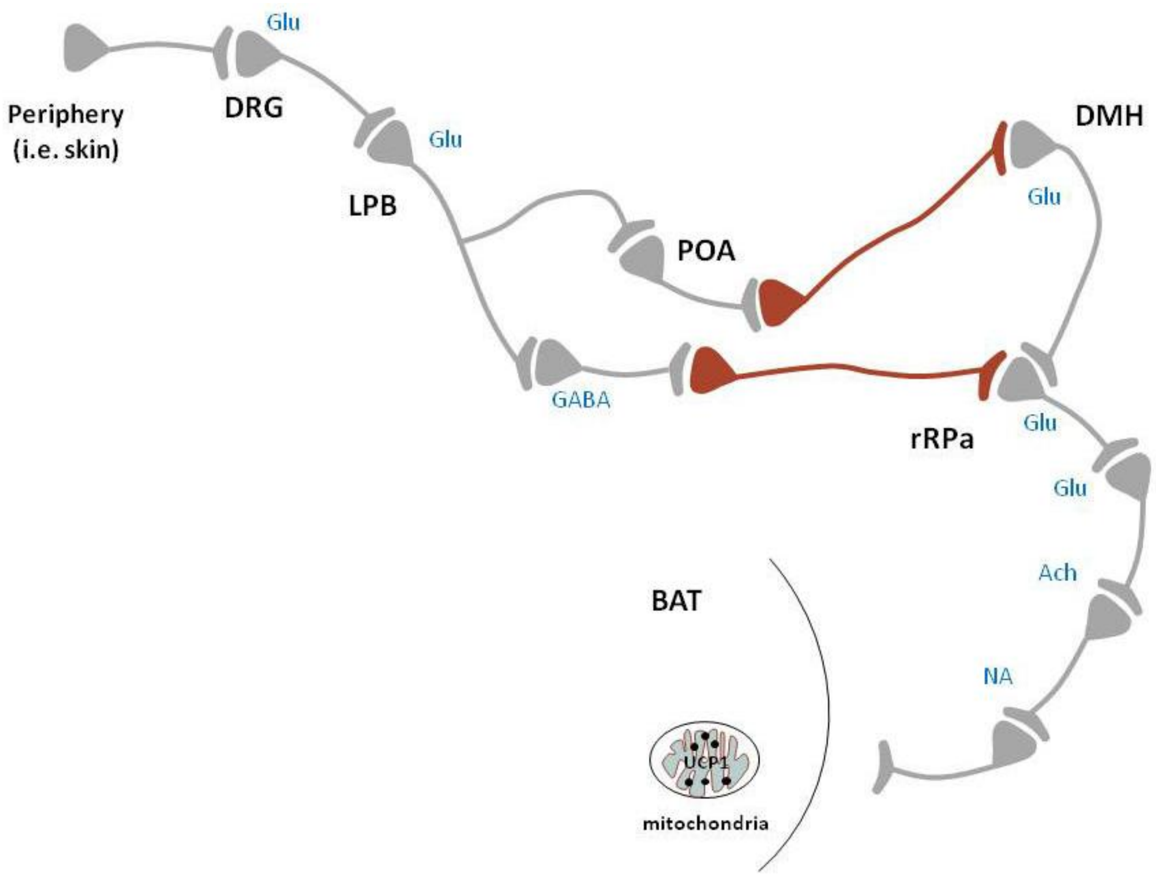

**FIGURE 4.** Schematic representation of the neuronal pathways involved in temperature regulation and the fever response. Cold-, warm-, and pyrogen-sensitive GABAergic projection neurons (highlighted in red) are part of a neuronal pathway that receives information about peripheral temperature and can regulate fever response by activating thermogenesis in BAT via UCP1. These neurons show the same changes in activity upon cold signals and pyrogens. Abbreviations: BAT, brown adipose tissue; DMH, dorsomedial hypothalamus; DRG, dorsal root ganglion; LPB, lateral parabrachial nucleus; POA, preoptic area, rRPA, rostral raphe pallidus. Glu, glutammate; GABA, gamma amino butyric acid; Ach, acetylcholine; NA, noradrenaline; UCP1, uncoupling protein 1.

## CHEMISTRY AND PHARMACOLOGY OF FEVER

The cascade of events that triggers the fever response is mediated by a remarkable variety of chemical reactions and molecular interactions. We summarize some of the most significant ones.

Fever can be initiated when high-affinity (nanomolar Kd or higher) recognition of an exogenous (bacterial, viral) or endogenous agonist molecule occurs via TLRs. This recognition event permits a limited number of molecules to activate the signaling pathway leading to NF-κB–dependent gene transcription of endogenous pyrogens, such as IL-1β (Fig. 5). We estimated that binding of one LPS molecule from a Gram-negative bacterium can give rise to approximately 10,000 IL-1β molecules secreted by the macrophage following activation of TLR4. The signal is then further amplified as secreted IL-1β activates other macrophages as well as T and B cells of the humoral immune response and the fever response.

Receptor dimerization is an important element in the activation of the TLR signaling. In fact, binding of the receptor to its ligand is followed by heterodimerization of the receptor and accessory protein via the 200-amino-acid-long TIR domain[20]. Crystallographical analysis of the TIR domain identified a specific region (known as the BB loop) as being critical for TIR-TIR dimerization[20]. This critical step was the target for a rational drug design of small molecules that could inhibit its formation. Rebek and colleagues synthesized BB-loop mimics of monomeric[49] and dimeric[50] versions, connected by a linker (Fig. 6). These small molecules, like AS-1 and EM63, have been shown to block the Toll signaling *in vitro* and *in vivo*.

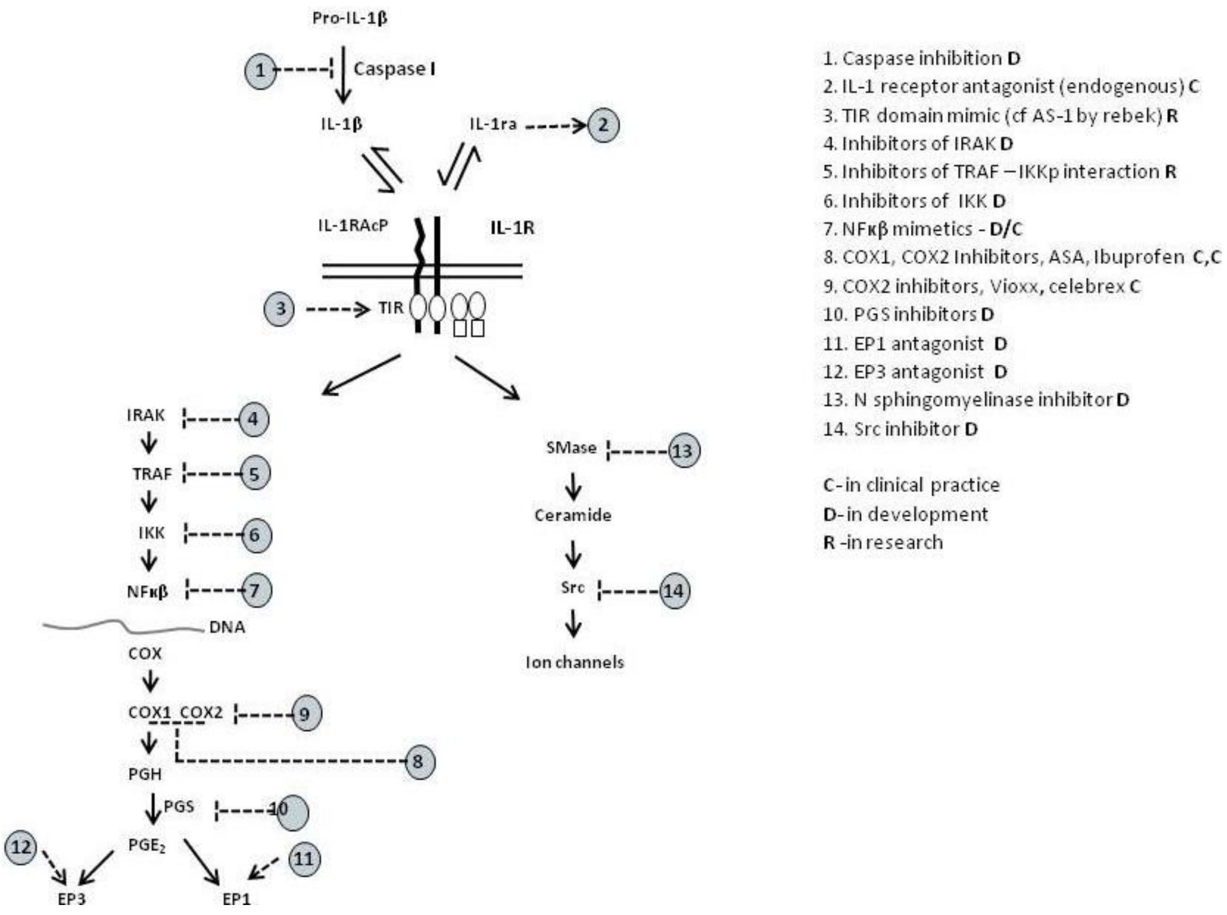

**FIGURE 5.** The sites of action of antipyretics in clinical practice and in development. Schematic representation of the site of action of antipyretic drugs in clinical practice, in development, or still being investigated.

Toll signaling also includes multiple serine-threonine and tyrosine phosphorylations. Toll receptors, like IL-1R1, are phosphoproteins that are phosphorylated by Src-type soluble tyrosine kinases. Protein phosphatases are, of course, an integral part of the Toll signaling system, resetting the system to be able to respond anew.

Possibly the single, most relevant pharmacological contribution to fever research was the chemical synthesis of acetylsalicylic acid (Fig. 2). First synthesized by Charles Frédéric Gerhardt in an unstable form and finally by Felix Hoffman in 1899 in its stable form, it was patented and marketed by Bayer with the name aspirin. Aspirin remains the most well-documented drug, taken in the largest number of doses, and by the largest number of people, and is also recommended and widely used as a preventive cardiovascular drug to reduce clotting, myocardial infarction, and stroke[51]. Its introduction contributed to both a decline in the interest of fever research, for fever could now be controlled, as well as to an increased interest in using this molecule to gain insights into an understanding of the mechanisms of fever. Among the puzzling question was how aspirin could reduce CBT during fever, but have no effects on the basal CBT when the same dose is taken by the same individual to ease a headache.

Production of endogenous pyrogens like PGE2 proceeds from arachidonic acid via several enzyme-catalyzed steps, of which the mixed function oxygenase, the constitutively expressed cyclo-oxygenase 1, and the inducible cyclo-oxygenase have been targets of anti-inflammatory and antipyretic drugs, commonly labeled as nonsteroid anti-inflammatory drugs (NSAIDs). Inhibitors of COX1 and 2 include acetylsalicylic acid, ibuprofen, and the COX2-selective inhibitors: Vioxx, Bextra, and Cerebrex. The latter three were rationally designed enzyme inhibitors[52,53,54].

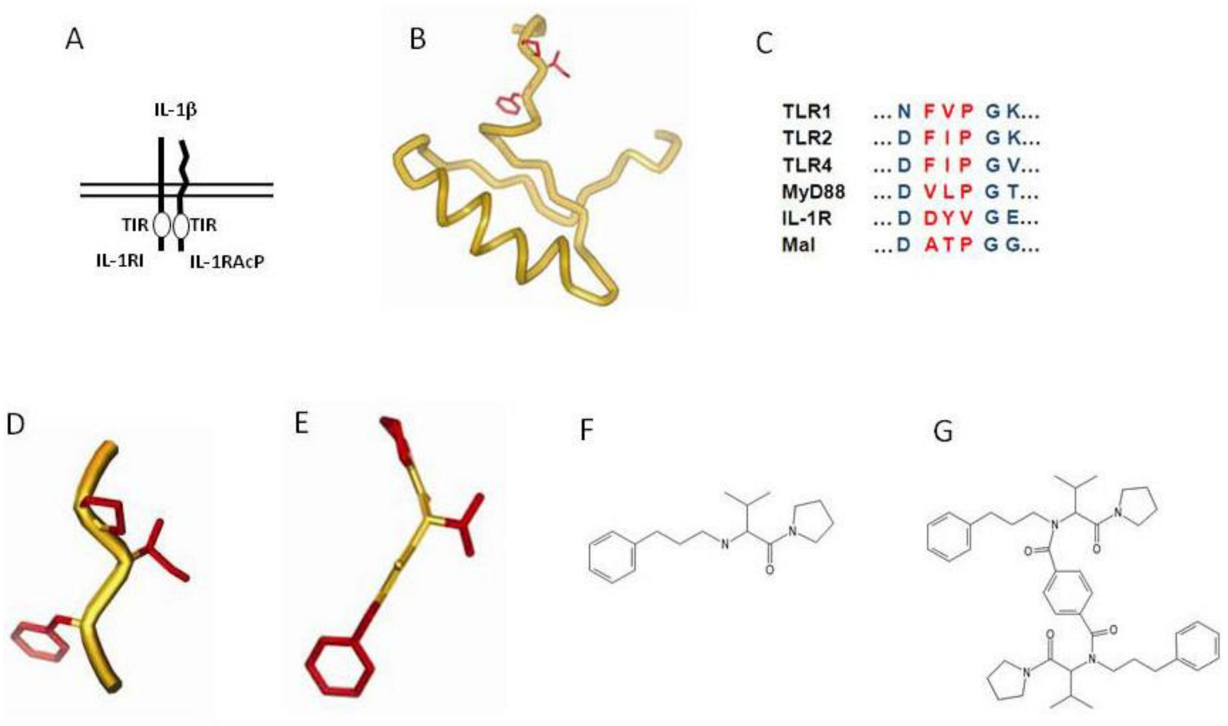

**FIGURE 6.** Rational design of inhibitors of the protein-protein interaction central to Toll signaling in fever and recognition of microbial products. The TIR domains found in the IL-1R1/AcP (and in the TLRs) interact via their BB loop (A). A model of the BB loop derived from the resolved crystal structure of the TIR domain (B). The synthesis of BB-loop mimics was based on the [F/Y-V/L/I-/P/G] consensus sequence found in the TIR domains of TLRs, IL-1R1, MyD88, and Mal (C). A detail of the BB-loop model (D) was used to synthesize the monomeric BB-loop mimic AS-1 (E and F). A dimeric/bifunctional BB-loop mimic, EM77, that "keeps apart the two BB domains" was also synthesized (G). AS-1 and EM77 were synthesized by the Rebek laboratory at The Scripps Research Institute.

The irreversible acetylation of COX by acetylsalicylic acid has been much studied[55], and it is ironic that aspirin, an irreversible enzyme inhibitor, is one of our most used drugs, prescribed for a large number of conditions to children and adults alike. Nevertheless, its usefulness is undisputed as an NSAID and as an inhibitor of platelet aggregation and its price at the pharmacy is unbeatable.

We have not discussed the strong anti-inflammatory and antipyretic effects of steroid drugs, all of which down-regulate the entire IL-1 system at the level of gene transcription. Steroid therapy–mediated suppression of inflammation involves suppression of febrile response by the endogenous inflammatory pyrogens[56]. Yet because of the multiple side effects of steroids, these are not indicated for suppressing fever.

Because of the high amplification of the signal through Toll receptors that include the IL-1 receptor itself, pharmacological activation of the cascade is best achieved at the top of the reaction cascade. Similarly, blockade of IL-1 and Toll signaling is most effective at the top of the signaling cascade by blockade of the receptor, as Nature practices it, i.e., by the endogenous IL-1 receptor antagonist. It should also be noted that many microbial agents have decoy proteins that block Toll receptors or TIR-TIR interactions required for Toll signaling.

The conservation and production of heat during the fever response is not discussed here although these processes, in a coordinated fashion, contribute to the elevation of the CBT. Vasoconstriction in the extremities and the adaptive thermogenesis, heat production by shivering, and, more importantly, by activation of brown adipocyte thermogenesis are important in achieving elevated temperature during fever in both infants and adults.

## SUMMARY

The molecular and biochemical mechanisms leading to synthesis and release of the endogenous pyrogens, IL-1 and PGE2, are now understood. The enigma of how chemically different bacterial or viral exogenous pyrogens cause the same stereotypic rise in CBT has been solved recently by the discovery of Toll receptors in macrophages. Distinct members of this receptor family recognize different and specific microbial components, but induce the biosynthesis and release of the same endogenous pyrogens, such as IL-1β, TNF, and IL-6. These cytokines regulate each other's production and stimulate the production of PGE2 that affects the central set point via the PG receptor EP3. Inhibiting the activity of COX1 and 2, the key enzyme in the biosynthesis of PGE2, with acetylsalicylic acid or ibuprofen, is the most widely practiced way to reduce fever. Work on the mechanisms of central thermoregulation identified the POA of the hypothalamus as one of the most important regions for the regulation of temperature homeostasis. This region contains temperature-sensitive neurons that respond to either an increase or a decrease in local temperature and are thus referred to as warm sensitive (WSN) or cold sensitive (CSN) neurons, respectively. Electrophysiological characterization taught us that pyrogens act on these specialized cells by inhibiting their activity. Thus, fever can be seen as resulting from the disinhibition of thermogenesis. Recent attempts at the comprehensive molecular characterization of WSN will likely further advance our understanding of the central mechanisms of temperature regulation and fever.

Fever remains one of the most reliable hallmarks of disease. The view of fever as part of generalized stress response has placed it firmly in the realm of physiology and we no longer treat it as solely a pathophysiological phenomenon.

The antipyretic drug arsenal is increasing in complexity with novel biological agents entering clinical practice, but for the most common fever, acetylsalicylic acid and ibuprofen, with their long safety track record and low price, are unbeatable as antipyretics of choice. Certain, hereditary febrile conditions, however, require the use of biologicals, such as IL-1RA, that block fever in the Mediterranean recurring fever syndrome and in Muckle-Wells disease.

The investigation of the molecular mechanism of action of pyrogen signaling via Toll receptors has brought a broad understanding of not only the coupling between exogenous and endogenous pyrogens, but also of how viral and bacterial products of great diversity can activate the innate immune system stimulating the release of endogenous pyrogens. The recognition that cytokine messengers in the immune system, such as IL-1, can also act on the central nervous system is the basis of work in the growing field of neuroimmunology. The heat production modes employed by homeotherms point to the enormous importance of mitochondria that couples metabolism and respiration, and as a generator of heat by uncoupling the energy reserves stored in the proton gradients. Fever studies on heat production and on regulation of CBT have given insights into the coupling between metabolism and energy utilization in metabolic diseases and in the process of aging. The synthetic chemical work that has been aimed at the blockade of the PGE synthesis and Toll signaling is an important part of the early development and of continued function of the pharmaceutical industry. Fever has proven to be and continues to be a significant source of new insights into biology and chemistry.

## ACKNOWLEDGMENTS

The authors wish to acknowledge important discussions with Lars Ernster, Barbara Cannon, Jan Needergard, Charles Dinarello, and Clifford Saper that helped us to maintain focus and enthusiasm for the topic during the years. We are indebted to our many collaborators over the years. We thank Drs. Brad Morrison and Olivia Osborn for their kind assistance in preparing this manuscript. Supported by The Ellison Medical Foundation, NIA AG028040 and The NIH 5 R01 MH074055-03 and 1 U01 NS063560-01.
